# An In-Vitro Study on the Impact of Light-Emitting Diode (LED) and Laser-Activated Bleaching Techniques on the Color Change of Artificially Stained Teeth at Varying Time Intervals

**DOI:** 10.7759/cureus.69851

**Published:** 2024-09-21

**Authors:** Taniya Elsa Oommen, Aishwarya Arya, Bisma Jahangeer, Divya Mishra, Kanuri Venkta Naga Vamseekrishna, Jatin Gupta

**Affiliations:** 1 Department of Conservative Dentistry and Endodontics, Private Practitioner, Kerala, IND; 2 Department of Conservative Dentistry and Endodontics, Awadh Dental College and Hospital, Jamshedpur, IND; 3 Department of Conservative Dentistry and Endodontics, Private Practitioner, Kulgam, IND; 4 Dentistry, Heritage Hospital, Varanasi, IND; 5 Department of Conservative Dentistry and Endodontics, MM College of Dental Sciences and Research, Mullana, IND; 6 Department of Conservative Dentistry and Endodontics, Care Dental College, Guntur, IND; 7 Department of Orthodontics and Dentofacial Orthopaedics, Private Practitioner, Haryana, IND

**Keywords:** 35% hydrogen peroxide, artificially stained teeth, color change, cosmetic dentistry, dental aesthetics, in-office bleaching, laser activation, led activation, tooth bleaching, tooth whitening

## Abstract

Introduction

Tooth color is a key factor in the perception of an attractive smile, making bleaching a widely sought-after cosmetic dental procedure. Valued for its minimally invasive approach, this technique effectively lightens teeth to achieve desired aesthetic outcomes. As the desire for immediate results grows among patients, various bleaching agents, and energy sources have been developed to expedite the bleaching process. The study aims to evaluate and compare the efficacy of 35% hydrogen peroxide activated by light-emitting diode (LED) and laser on artificially stained teeth, assessing color changes at different time intervals.

Methodology

Sixty maxillary central incisors with intact crowns were selected for the study. An artificial staining solution was prepared, and the samples were immersed in it for 15 days to simulate staining. After this period, the teeth were removed from the solution, rinsed with water, and dried. Baseline photographs of each tooth were taken for comparison. The teeth were then divided into two groups, one treated with LED activation and the other with laser activation of 35% hydrogen peroxide as the bleaching agent. The color changes were measured and recorded at different time intervals. Data were presented as mean and standard deviation, with statistical analysis conducted using independent sample t-test and repeated measures ANOVA to compare the efficacy of the two methods.

Results

Both LED and laser activation of 35% hydrogen peroxide demonstrated significant color changes in the stained teeth. The initial color improvement was noticeable in both groups, with LED and laser treatments producing comparable whitening effects. The mean color change values indicated that both activation sources were effective in enhancing the bleaching process, providing immediate and substantial results. Statistical analysis showed no significant difference between the two methods, suggesting their equivalency in clinical efficacy for tooth bleaching.

Conclusion

The study concludes that both LED and laser activation of 35% hydrogen peroxide are efficient methods for in-office tooth bleaching, providing immediate and enhanced whitening effects. The comparable results between the two energy sources indicate that can be effectively used to meet the increasing demand for rapid aesthetic improvements in a clinical setting. This finding supports the adoption of both LED and laser bleaching techniques as viable options for dental practitioners aiming to achieve optimal patient satisfaction with minimal invasiveness.

## Introduction

It is widely acknowledged that in today's society, appearance plays a significant role, with a smile being one of the most impactful factors in enhancing one's overall appearance. Increased awareness and improved self-esteem have led people to shift their focus towards aesthetics [1,2]. The color of teeth is a crucial parameter for a beautiful smile, influenced by factors such as intrinsic and extrinsic stains, dentin exposure, gum recession, enamel translucency, and the morphology and thickness of enamel [3]. Several techniques have been developed to improve tooth color, including professional scaling and polishing, whitening toothpastes, crowns, and veneers, bleaching of both vital and non-vital teeth, and enamel micro-abrasion using abrasives and acids [4].

Among these methods, tooth bleaching is the most commonly utilized technique due to its relatively non-invasive nature, quick results, and cost-effectiveness. The bleaching process works by breaking down conjugated bonds within stain-associated protein chains (chromatophores) through the oxygen released from hydrogen peroxide, a potent oxidizing agent [5]. This chemical reaction enhances the absorption of color wavelengths, resulting in reduced color reflection and a whitening effect [6]. A 35% concentration of hydrogen peroxide is often used because it penetrates the enamel structure effectively. To cater to the growing demand for faster results, various energy sources have been employed to expedite the bleaching process [7]. These include heat, light sources such as (light-emitting diode) LED and halogen curing lights, plasma arcs, and lasers (diode and KTP lasers (potassium titanyl phosphate), as well as LEDs with lasers). These energy sources accelerate the decomposition of peroxide, increasing the release rate of bleaching radicals and achieving whiter teeth in a shorter period [8,9].

LED fixed appliances emit uniform light in the blue and violet wavelength range and are equipped with filters to avoid infrared radiation, thus lowering the pulpal temperature and providing energy for the bleaching reaction [10,11]. It has been claimed that hydrogen peroxide activation by diode laser not only provides a whitening effect but also prevents changes in enamel structure compared to bleaching without activation [11,12].

Using standardized shade guidelines such as Vitapan Classical and Vita Bleached Guide 3D Master6, color changes can be analyzed to evaluate the therapeutic outcome of different tooth whitening procedures [13]. Analyzing digital photos using commercial software has made determining bleaching effects straightforward and accurate. L*a*b* values, a color industry standard, are used to determine color based on high-resolution photos shot with digital SLR cameras using programs such as Adobe Photoshop Reader, and Adobe Systems Incorporated, San Jose, California [14,15]. Two main issues are the resulting shade's long-term stability and the possibility of a rebound impact after bleaching. Numerous in-vitro and in-vivo studies are actively debating how well various light-activating methods produce color change. Therefore, the purpose of this study is to assess and contrast the color change in artificially stained teeth that results from activating 35% hydrogen peroxide with a laser and LED at different time intervals.

## Materials and methods

Collection of sample

Ethical approval for this in vitro study was obtained from the Institutional Ethics Committee (IEC 1797) of the Maharishi Markandeshwar College of Dental Sciences and Research, Mullana, Ambala. The investigation was conducted in the Department of Conservative Dentistry and Endodontics. A total of 60 maxillary central incisors with intact crowns were selected, focusing on these anterior teeth due to their critical role in facial aesthetics. Central incisors are particularly important as they are the most visually prominent and often align with the golden proportion, making them central to cosmetic appeal. The sample size was calculated using G Power analysis, which determined that approximately 64 teeth are required to achieve a power of 80% with a medium effect size (0.5) and an alpha level of 0.05. Although the study uses 60 teeth, which is close to the estimated requirement, this sample size should provide sufficient power to detect a statistically significant effect if one is present. Inclusion criteria ensured that all selected teeth were extracted for orthodontic or periodontal reasons and exhibited no hypoplastic defects, caries, restorations, cracks, fractures, or discolorations. Teeth with compromised crown structure, carious or non-carious defects, fractures, cracks, hypoplastic defects, restorations, prostheses, or previous endodontic treatment were excluded from the study.

Preparation of samples

After using an ultrasonic scaler to clean the teeth samples' surfaces and eliminate any remaining tissue or extrinsic stains, the samples were submerged in a 0.5% chloramine-T solution for about a week to disinfect them. After that, the samples were kept in regular saline throughout the experiment. After a week, the labial surfaces of the teeth were cleaned with pumice paste (DPI, Propol prophylaxis paste) and a rubber cup (SHOFU, Japan).

**Figure 1:**
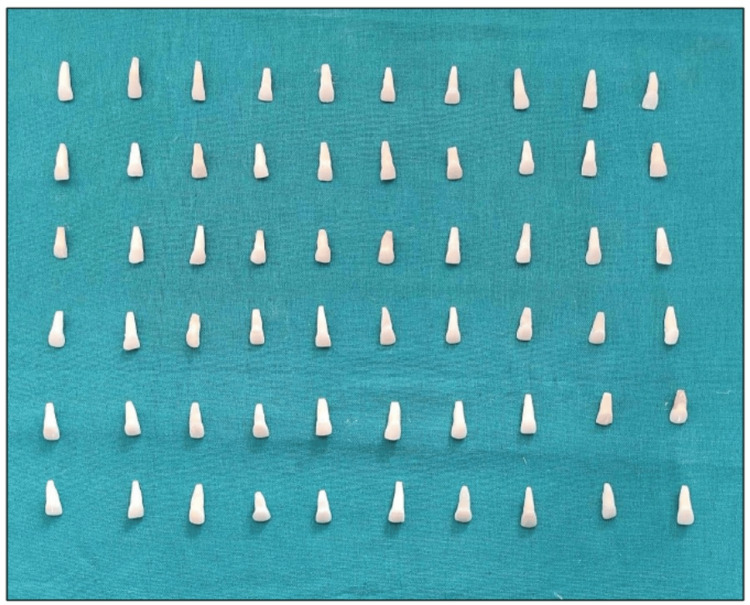
Sectioned samples of the teeth taken for the study

In order to induce internal staining, two millimeters (mm) of root tip were cut (Figure 1) and immersed in an artificial staining solution.

**Figure 2:**
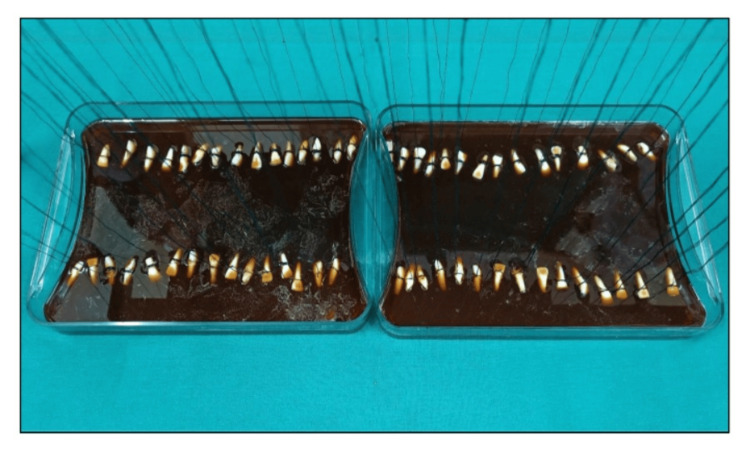
Samples submerged in an artificial staining solution

**Figure 3:**
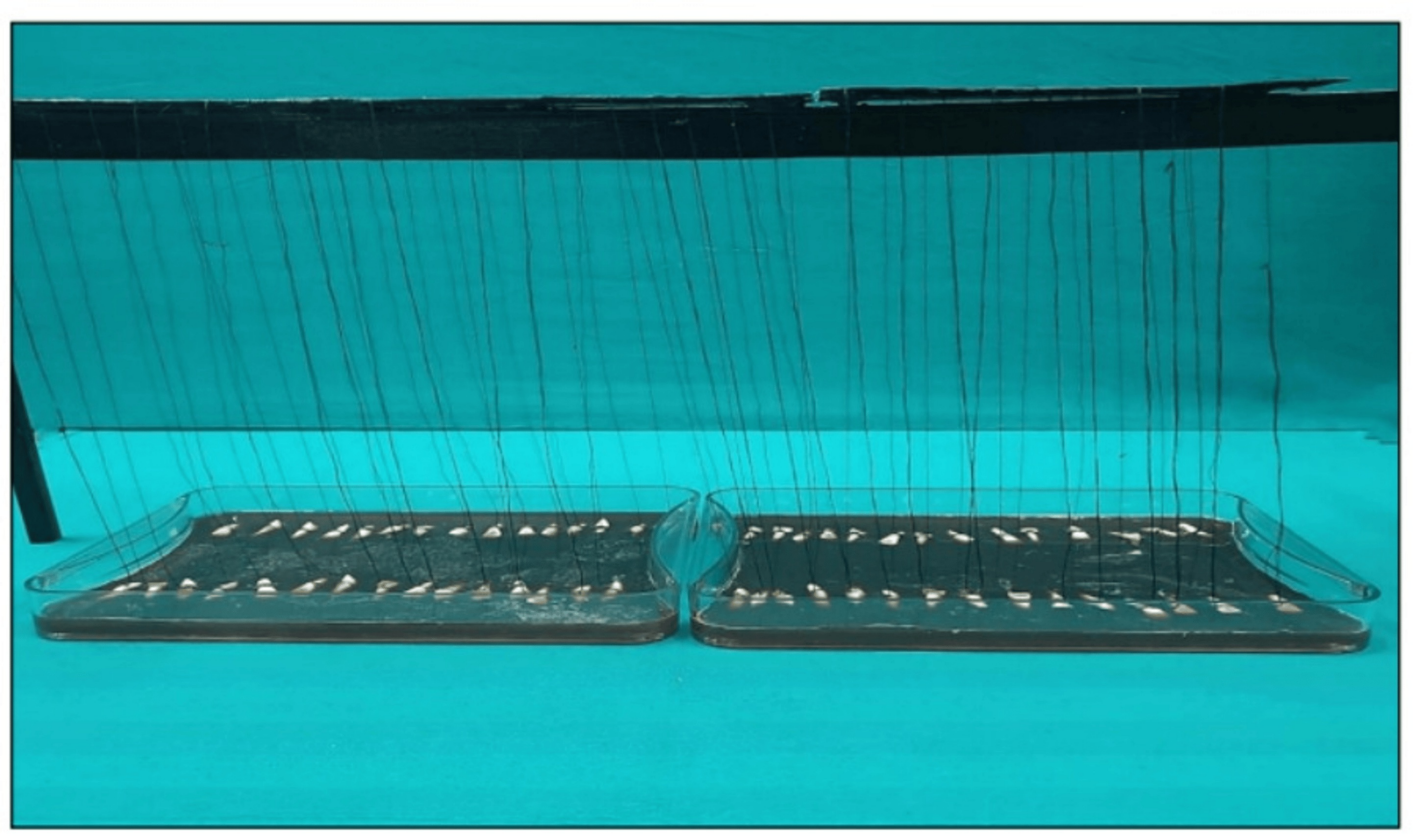
Samples hanging on a thread in the staining solution for staining

Each tooth was immersed in the solution in a vertical position with the help of a thread tied onto a customized stand such that the samples did not contact each other to allow the deposition of stains on the surface of the sample only (Figures 2, 3). The samples were rinsed with water and placed back after refreshing the solution every day for 15 days.

Preparation of an artificial staining solution

**Figure 4:**
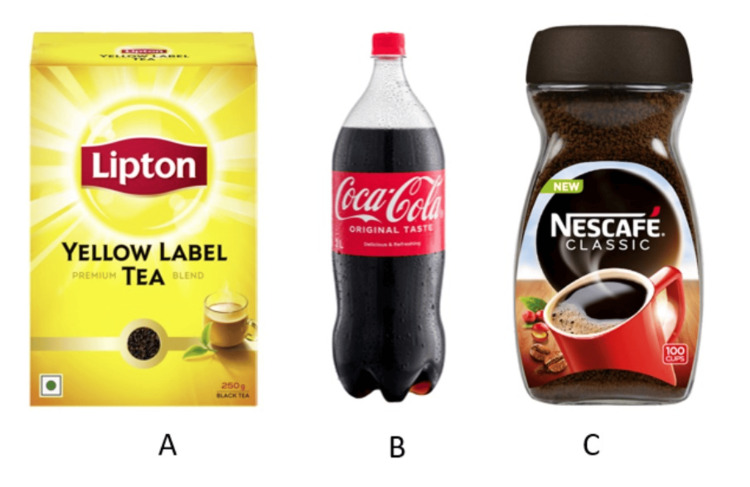
Materials for preparation of an artificial staining solution of tea (A: Lipton), Cola (B: Coca Cola) and coffee (C: Nescafe) Lipton: Rotterdam, Netherlands; Coca Cola: Atlanta, Georgia, USA; Nescafe: Orbe, Switzerland

**Figure 5:**
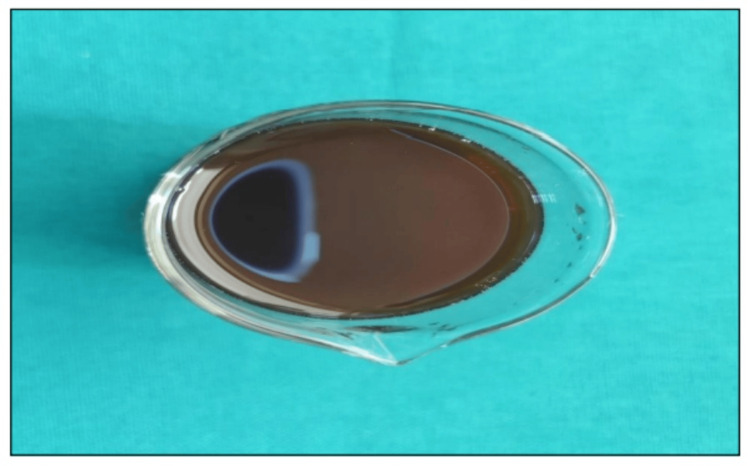
Final prepared solution of artificial staining solution

A synthetic staining solution was created by combining 100 ml of Coca-Cola (Atlanta, Georgia, USA), Nespresso (Vevey, Switzerland), and Lipton tea (Rotterdam, Netherlands), respectively. (To make the tea solution, immerse a tea bag in 100 milliliters of boiling water for five minutes; to make the coffee solution, place ten grams of coffee in the same amount of boiling water) (Figures 4, 5).

**Figure 6:**
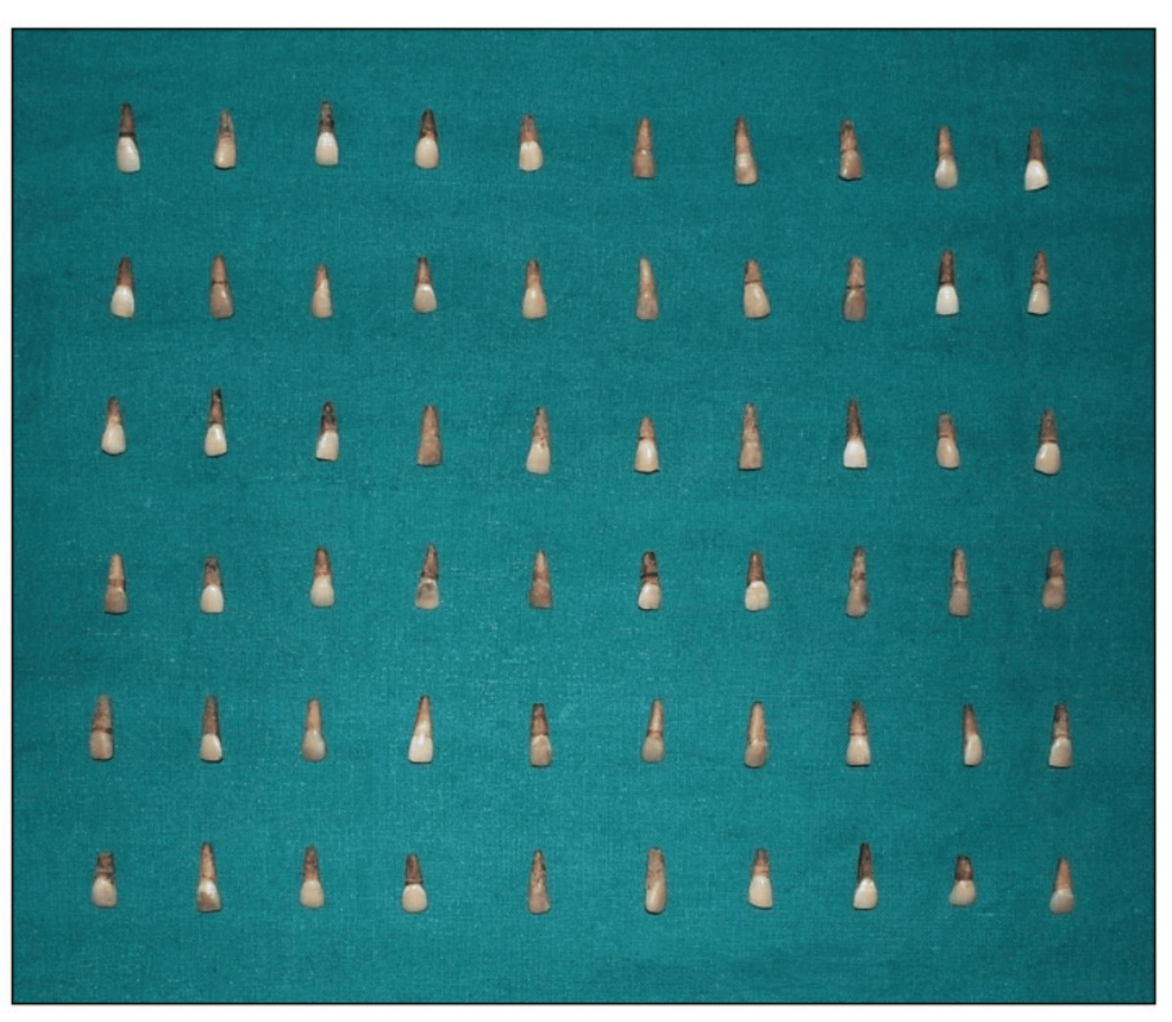
Artificially stained teeth samples

After 15 days, teeth were taken out of the staining solution and washed with water. Once the teeth had dried, baseline pictures of every tooth were captured (Figure 6).

The next step was to install each tooth on a wax block so that it could be bleached and its distance from the laser whitening and LED handpiece standardized.

Formation of groups

On wax blocks, the teeth were numbered from 1 to 60. Based on the manner of activation, the teeth were then split into 2 groups, Group A (LED activation) and Group B (laser activation), each with 30 samples. Based on the intervals at which color changes would be assessed, each group was then split into three subgroups of ten, namely a1, a2, and a3 for Group A as well as b1, b2, and b3 for Group B. For each group, the change in color will be assessed seven days, twenty-one days, and six months after bleaching.

Procedure

Group A

**Figure 7:**
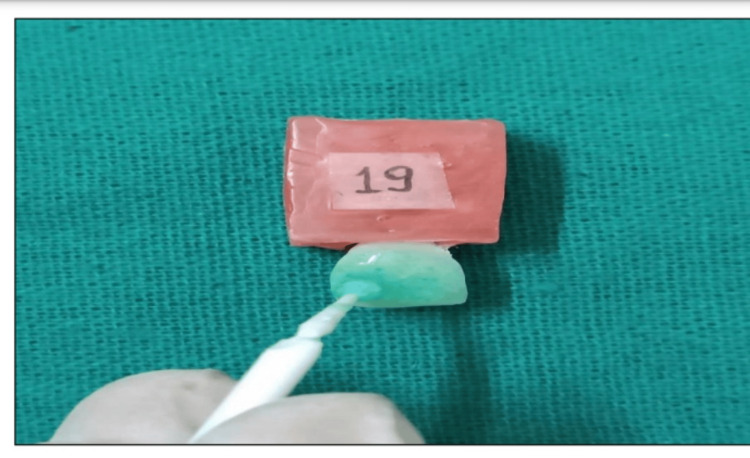
Application of bleaching gel on the sample

**Figure 8:**
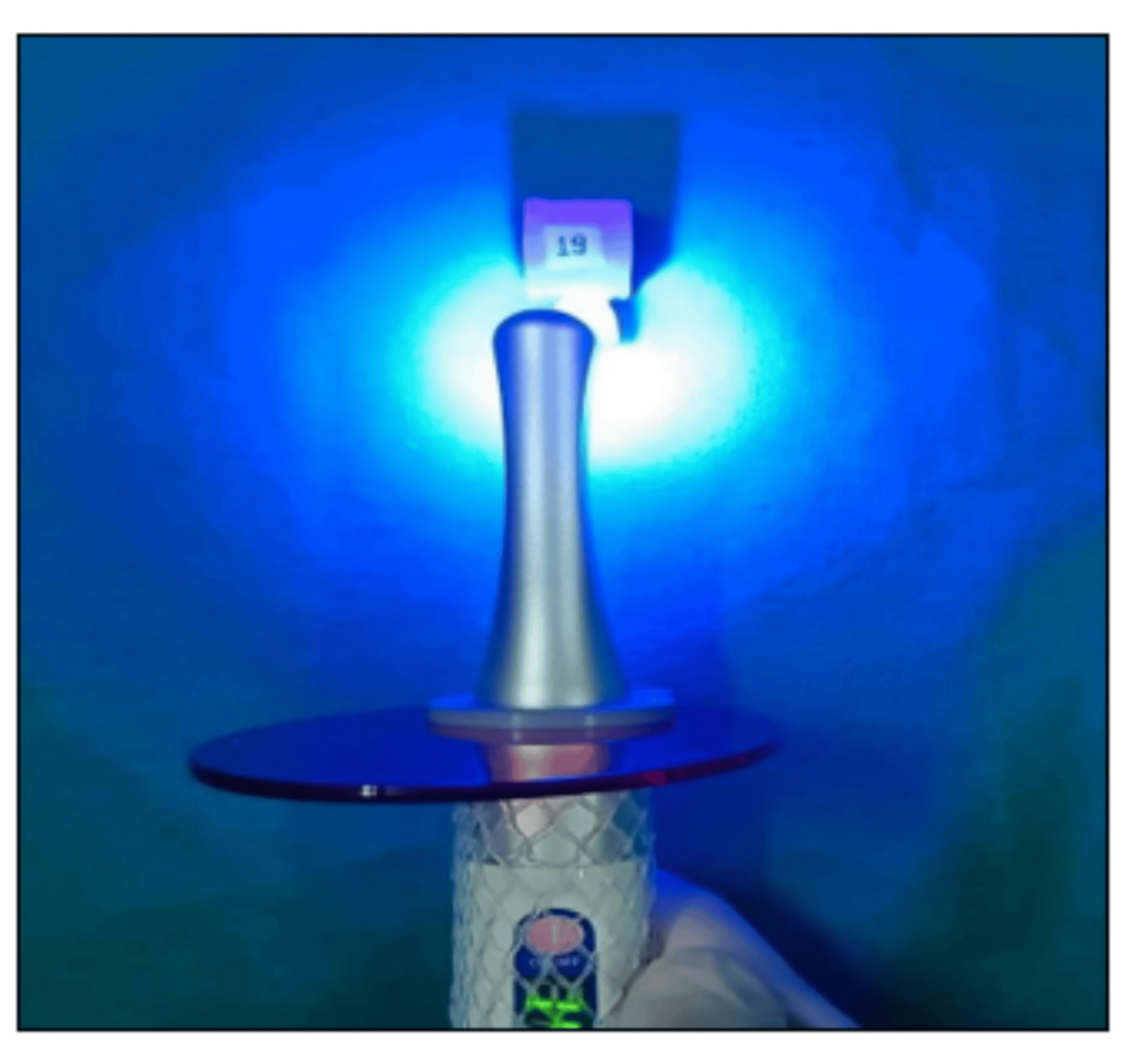
Activation of bleaching gel using woodpecker ILED plus curing light

As directed by the manufacturer, the contents of both the powder pot and syringe were mixed to create SDI Pola-Office whitening gel (35% hydrogen peroxide) at uniform room temperature. The labial surface of the Group A sample teeth received an even layer of gel (about 1-2 mm thick) for 8 minutes. The gel was then activated for 20 seconds using an LED light source that was positioned at a 45-degree angle to a line perpendicular to the tooth surface, covering the entire tooth surface with light (Figures 7, 8). The manufacturer's instructions were followed, and the process was done three times. After using a fresh gel to stain the samples every time, they were cleaned with a clean cotton swab, rinsed with water, and dried.

Group B

As directed by the manufacturer, the contents of the base and activator syringes were mixed to create Biolase (Foothill Ranch, California, USA) Laser White 20 gel (35% hydrogen peroxide) at room temperature.

**Figure 9:**
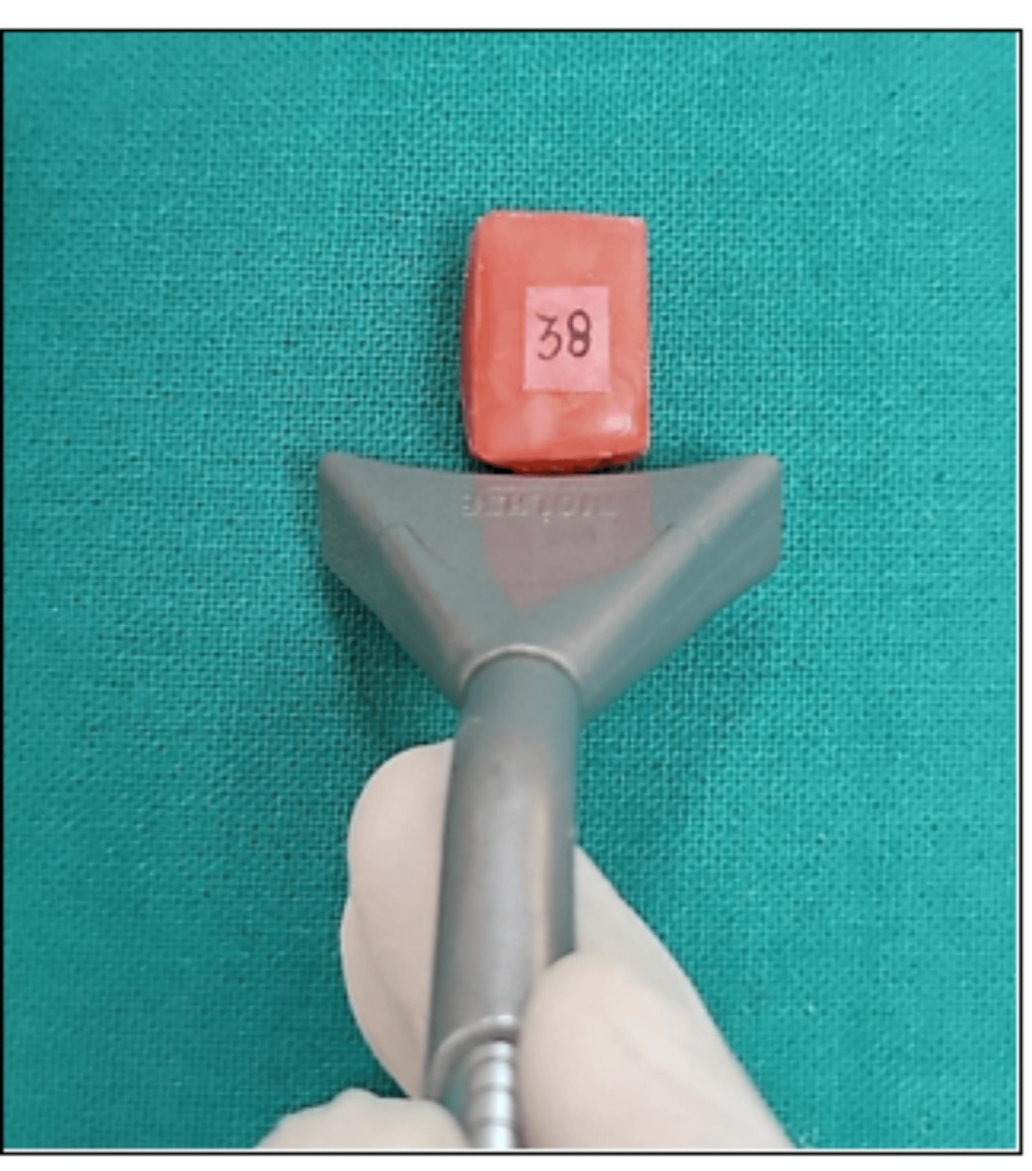
Activation of the bleaching gel by Biolase Epic X Diode Laser

Using an applicator tip, an even layer of Laser White 20 gel (about 1-2 mm thickness) was applied to the buccal surface of the teeth in the Group B sample. Then, for 30 seconds, they were exposed to radiation from a 7 W, 940 nm diode laser (Biolase Epic X [Foothill Ranch, California, USA]) at a distance of 1 mm. The laser's whitening handpiece was placed across a 1 cm2 area (Figure 9). To regulate the temperature rise, this method was done three times, with a 5-minute rest period in between each irradiation. They wiped off the gel.

Photographic record

**Figure 10:**
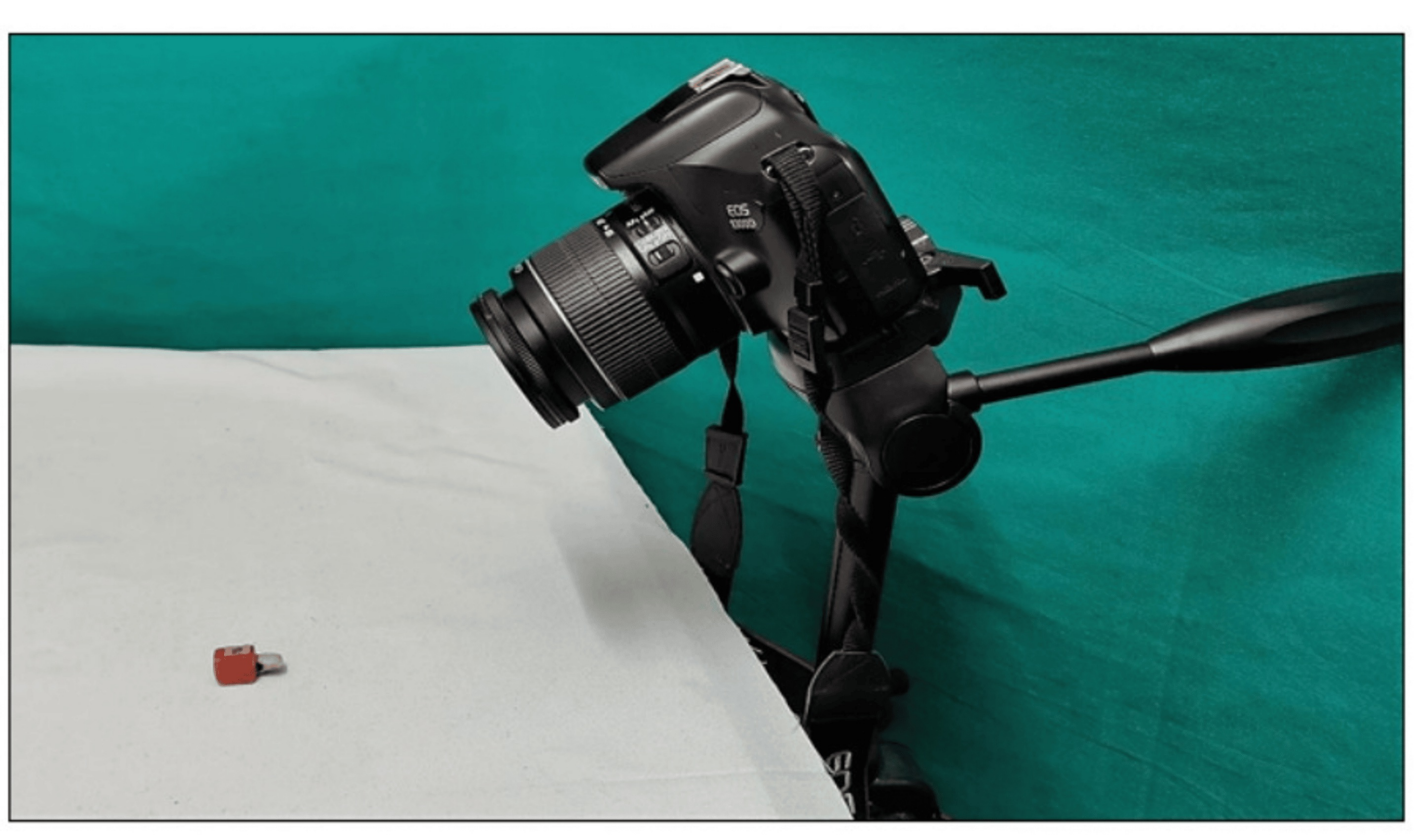
Camera setting for taking digital photographs

Before bleaching and after artificial staining, photos were taken (Figure 10). Post-bleaching photos were taken as soon as the bleaching was completed and at various intervals determined by the subgroups, namely seven days, 21 days, and six months for each group.

In a semi-dark environment, tooth samples were standardized and placed on a designated area over a white background. Using a DSLR camera (Canon EOS 1300D [Tokyo, Japan]) with the following settings: F22 aperture, 1/25 shutter speed, ISO 100, manual flash at ½ power, and 450 angulation, photos were taken of the buccal side of the tooth sample. To achieve angulation uniformity, the camera was mounted on a tripod stand.

After transferring all of the digital photos that had been shot at predetermined intervals to the computer, Adobe Photoshop 2021 (California, USA) was used to evaluate the color of each image, taking into account the shade of the teeth by examining a small circular area in the center. The Commission International de l'Eclairage's (CIE) L*a*b* system's protocols were used to record the color space. L* stands for lightness or luminance, which ranges from 0 [black] to 100 [white]. A* denotes the red-green axis, which is green-negative a* and yellow-positive b*. B* denotes the yellow-blue axis, which is blue-negative b*. The color change between the baseline and designated time intervals is represented by ∆E.

∆E = [(∆L) 2 + (∆a) 2+ (∆b) 2]1/2

**Figure 11:**
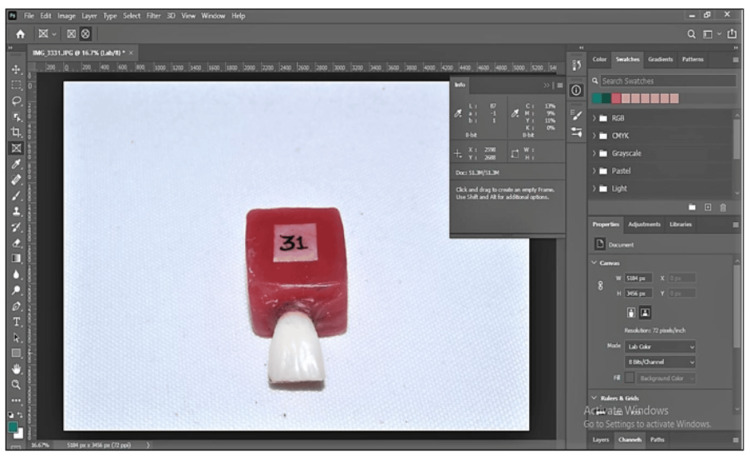
Evaluation of L* a*b values using Adobe Photoshop 2021

ⵠE value was calculated by Microsoft Excel 2016 (Redmond, USA) using this equation. The values were compared between the subgroups at specific time intervals for each group and analyzed simultaneously (Figure 11).

The gathered data was evaluated further by statistical analysis, and conclusions were drawn from the data using statistics. The mean and standard deviation of the data were displayed. The independent samples t-test was used to evaluate continuous variables, while the ANOVA test was used for repeated measures. Significance was assessed using a two-tailed test and p-values less than 0.05. IBM Corp. Released 2012. IBM SPSS Statistics for Windows, Version 21.0. Armonk, NY: IBM Corp. was used for data analysis.

## Results

Artificially stained teeth treated using 35% hydrogen peroxide activated by either LED or laser at varying intervals (seven days, 21 days, and six months) were the subject of this study, which aimed to compare and assess the color change (ΔE).

Comparative analysis between groups

At the first time interval, there was no statistically significant difference in the ΔE values between the LED (a1) and laser (b1) groups, according to the inter-group comparison (p=0.531). In a similar vein, at the second time interval, there was no discernible difference between the LED (a2) and laser (b2) groups (p=0.116). Nonetheless, during the third time interval, a statistically significant difference in ΔE values was noted between the LED (a3) and laser (b3) groups (p=0.044) (Table 1).

**Table 1:** Inter-group comparison of ΔE values between LED and laser treatments across three time intervals ANOVA test, p-values not statistically significant. F value is 1.298

Subgroups	Mean ± Standard deviation	p-value
a_1_	13.82 ± 4.22	0.531
b_1_	15.20 ± 5.41	
a_2_	13.16 ± 1.68	0.116
b_2_	14.69 ± 2.40	
a_3_	9.75 ± 2.65	0.044
b_3_	13.99 ± 5.61	

Intra-group comparison

For the LED group, the comparison of ΔE values across the three follow-up periods (a1, a2, a3) showed no significant difference (p=0.064) (Table 2). Similarly, the laser group also exhibited no significant difference in ΔE values across the corresponding follow-up periods (b1, b2, b3) (p=0.360) (Table 3).

**Table 2:** LED group comparison across intervals ANOVA test, p-value statistically not significant. F value is 4.554

Time interval	Mean +/- Standard deviation	p-value
a1	13.82 ± 4.22	0.064
a2	12.16 ± 1.68	
a3	9.75 ± 2.65	

**Table 3:** Laser group comparison across intervals ANOVA test; p-value statistically not significant. F value is 1.089

Time interval	Mean +/- Standard deviation	p-value
b1	15.20 ± 5.41	0.360
b2	14.96 ± 2.40	
b3	12.29 ± 5.61	

The results indicate that while both LED and laser activation are effective in bleaching, their efficacy in terms of ΔE varies over time. Specifically, the significant difference observed at the third time interval suggests that laser activation may have a more pronounced effect on tooth whitening at later stages compared to LED activation. Additionally, the intra-group comparisons demonstrate that there are no significant changes within each group over the follow-up periods, indicating consistent bleaching effects over time.

## Discussion

A smile is a reflection of one’s inner self and a bright smile that is in perfect harmony with the face and lips accentuates the charm of one’s character [15]. Regardless of age, patients frequently express a desire to have whiter teeth. Many patients experience an aesthetic issue with discolored teeth, especially in the maxillary anterior region [16]. Bleaching is a process that uses a chemical substance to change the tooth material's ability to reflect or absorb light by improving the appearance of brightness and oxidizing the discolored areas of the tooth structure. However, it should be noted that generally, the application of bleaching agents multiple times is required to get the desired aesthetic results, leading to a lengthy course of treatment [17,18]. New systems have since been created that use light to accelerate the whitening process by promoting the dissociation of the hydrogen peroxide while producing less heat, such as plasma arc halogen curing lights, xenon-halogen lights, lasers, light-emitting diodes (LEDs), and LED plus lasers. Light-emitting diodes (LEDs), especially blue LEDs, and diode lasers are some of the more recent irradiation technologies in photo-assisted bleaching, and it is regarded as the largest advancement in aesthetic dentistry in the last 10 years [19]. According to Ferrarazi et al., LED lamps provide a reliable, secure, and affordable way to activate hydrogen peroxide [19]. Dostalova T. et al. stated that the duration of bleaching can be shortened without surface changes using selective diode laser light [20].

There are numerous techniques used to assess tooth shade changes during bleaching; the CIEL*a*b color system is a significant accomplishment in this endeavor. Researchers have begun to employ digital cameras to evaluate bleaching methods by creating a digital image and uploading it to image-editing software (for instance, Adobe Photoshop from San Jose, California-based Adobe Systems Incorporated) [11]. In addition to saving time and a cost-effective method, photographs can offer a more permanent baseline record. The rebound effect following teeth whitening is a phenomenon linked to the reversal of tooth whitening and is the most unreliable feature of bleaching in its long-term stability. The controversial results among the published results of in-office bleaching using LED and laser-activated bleaching make further research necessary. Hence, our study evaluated the effect of LED and laser-activated bleaching techniques on the color change of artificially stained teeth at different intervals of time [21].

An artificial staining solution was prepared using tea, coffee, and Coca-Cola according to a method described by Saeedi R et al. to mimic the natural staining of teeth as they are the most commonly consumed beverages [8]. The chromogens of these drinks are usually questioned in a way that they cause only extrinsic discoloration until and unless the dentin is exposed. Hence, in this study, 2 mm of the roots of the samples were sectioned to cause intrinsic discoloration [11]. It was believed that enamel dehydration may have contributed to the first whitening of the tooth color [12]. Hence, to avoid the effect of enamel dehydration, the samples were stored in artificial saliva until the experiment to maintain rehydration. The teeth were then mounted on wax blocks to stabilize the samples during beaching as well as to standardize the distance of the samples from LED and laser handpieces [8].

In our study, the LED light system investigated was a blue LED by Woodpecker (ILED Plus) (Mumbai, India) with a wavelength range of 385 to 515 nm and an intensity of 1250 mW/cm². The laser system used was a diode laser Epic X by Biolase, a fourth-generation laser with a wavelength of 940 nm and a power of 7 W in continuous activation mode. Both groups used the same concentration of bleaching agent, 35% hydrogen peroxide. The LED group used the Pola Office gel (35% hydrogen peroxide, SDI), while the laser group used the Laser White gel by Biolase.

Matos LF, Hernandez LM, and Abreu indicated that significant changes in tooth color from bleaching are usually observed by the seventh day [5], while Wetter NU et al. reported color regression after 21 days post-bleaching [17]. Most studies suggest that the longevity of both at-home and in-office bleaching treatments is up to six months [12]. Our results showed no significant difference in the mean ΔE values between the LED and laser groups at both seven days and 21 days (p > 0.05). This finding aligns with Tekce AU et al., who found no significant difference between LED and laser-activated bleaching after one week and one month [14], and Marson FC et al., who also reported no significant color change from one week to one month post-bleaching [14]. However, our results differed from Rosentiel et al., who observed color relapse seven days after treatment with 35% hydrogen peroxide [22].

Our study revealed statistically significant color regression after six months, with the laser group showing less rebound compared to the LED group. This could be attributed to the higher ΔL value observed in the diode laser group at six months compared to the LED group [17]. The longer activation duration and greater wavelength of the diode laser may contribute to this difference [14,18]. The moderate color loss may result from the reformation of chemical bonds disrupted by the bleaching agents [15], as suggested by Lysons and Ng since the teeth were only held in saline and not exposed to any coloring agents. In LED power bleaching, color regression may occur due to remineralization alone, with delayed color assessment potentially misrepresenting the results [12].

These findings are consistent with Moghadam FV et al., who found no significant difference in color change up to three months but observed significant regression after six months [12], and Marson FC et al., who also noted significant color regression after six months [14]. When examining the LED (Group A) and laser (Group B) groups separately at seven days, 21 days, and six months, both groups initially exhibited effective color change, which gradually decreased, though the decrease was not statistically significant at any time interval.

This suggests that light activation using both LED and laser enhances whitening efficacy temporarily but does not maintain it long-term. Alomari et al. found that light acceleration only temporarily boosted the efficacy of bleaching agents and had no impact on long-term outcomes [18], while Hahn P et al. concluded that light-activated bleaching was ineffective for color stability beyond three months [11]. Therefore, within the limitations of our in-vitro study, both diode laser- and LED-activated bleaching systems showed comparable and satisfactory results for whitening extrinsic stains, but their color stability after six months was questionable. Further research is needed to confirm these results in clinical settings.

Limitations

This study, being an in-vitro investigation, did not simulate the complexities of the oral environment, thus failing to account for factors such as the remineralizing efficacy of saliva and the potential for further pigmentation from stains in food and beverages consumed post-bleaching. Additionally, the study could have benefited from a larger sample size and a longer evaluation period extending beyond six months to better assess the durability of the bleaching effects. The absence of color evaluation between 21 days and six months made it challenging to determine the precise timing of color regression.

## Conclusions

Within the limitations of this in-vitro study, it can be concluded that both LED and laser bleaching have produced enhanced whitening, which was comparable, but the color change was not sustained for a longer period of time and can be used as an efficient activation sources of bleaching agents for in-office bleaching in the clinical scenario for immediate results. The laser group has shown lesser regression of color at 6 months when compared to LED.
